# Persistent Opioid Use Associated With Dental Opioid Prescriptions Among Publicly and Privately Insured US Patients, 2014 to 2018

**DOI:** 10.1001/jamanetworkopen.2021.6464

**Published:** 2021-04-16

**Authors:** Kao-Ping Chua, Hsou-Mei Hu, Jennifer F. Waljee, Romesh P. Nalliah, Chad M. Brummett

**Affiliations:** 1 Susan B. Meister Child Health Evaluation and Research Center, Department of Pediatrics, University of Michigan Medical School, Ann Arbor; 2 Department of Health Management and Policy, University of Michigan School of Public Health, Ann Arbor; 3 Michigan Opioid Prescribing Engagement Network, Ann Arbor, Michigan; 4 Section of Plastic Surgery, Department of Surgery, University of Michigan Medical School, Ann Arbor; 5 University of Michigan School of Dentistry, Ann Arbor; 6 Division of Pain Medicine, Department of Anesthesiology, University of Michigan Medical School, Ann Arbor

## Abstract

This cohort study uses data from 3 MarketScan databases to compare the association of persistent opioid use with dental opioid prescriptions among publicly and privately insured patients in the United States from 2014 through 2018.

## Introduction

Persistent opioid use (POU) occurs when patients who receive opioid prescriptions after procedures continue to use opioids after acute pain typically resolves.^1,2,3^ In dentistry, the risk of POU has been assessed among privately insured patients.^2,3^ Whether the risk of POU differs among publicly insured patients is unknown. The goal of this study was to compare the risk of POU among privately and publicly insured dental patients aged 13 to 64 years in the United States.

## Methods

This retrospective cohort study used data from the 2014-2018 IBM MarketScan Dental, Commercial, and Multi-State Medicaid research databases. The Dental database contains dental claims from 1 million to 1.5 million nonelderly patients with employer-sponsored dental insurance; most claims can be linked to medical and pharmacy claims in the Commercial database. The Multi-State Medicaid database includes dental, medical, and pharmacy claims from 10 million to 12 million patients in several unidentified states. Because the data were deidentified, the University of Michigan Institutional Review Board deemed this study exempt from institutional review board review and patient informed consent. The study followed the Strengthening the Reporting of Observational Studies in Epidemiology (STROBE) reporting guideline.

Patients aged 13 through 64 years who had dental procedures between July 1, 2014, and December 31, 2017, were included in the study. Analyses were limited to each patient’s earliest procedure, the date of which was the index date. We excluded the following groups of patients: those lacking continuous enrollment during the 180 days before through 365 days after the index date, those who were not opioid naive, those who had dental procedures before the index date, and those who had subsequent surgical or dental procedures.

The exposure variable was set to 1 if there were 1 or more dispensed opioid prescriptions between 7 days before and 3 days after the index date (initial prescription).^2^ Persistent opioid use was defined as 1 or more dispensed opioid prescriptions 4 to 90 days after the index date and 1 or more prescriptions 91 to 365 days after the index date.^2^ Using logistic regression, we modeled the occurrence of POU as a function of the exposure, payer type, and their interaction. Models controlled for demographic and clinical characteristics. We calculated the average marginal effect (AME) of the exposure—the change in risk of POU if all patients did and did not have initial prescriptions—overall and by payer type. SAS version 9.4 (SAS Institute Inc) and Stata 14.2 MP (StataCorp) were used for statistical analysis. Two-sided hypothesis tests were conducted with α = .05.

## Results

A total of 1 691 878 patients were included in the study sample (Figure). Among these patients, 934 883 (55.3%) were female, 756 995 (44.7%) were male, and the mean (SD) age was 34.7 (16.3) years (Table). Among these patients, 38.5% were aged 13 to 25 years, and 37.0% were publicly insured; almost a third of patients (31.3%) had 1 or more initial prescriptions. The risk of POU was 1.3% overall and 2.1% vs 1.0% among those with and without 1 or more initial prescriptions (AME, 1.5 percentage points; 95% CI, 1.5-1.6 percentage points). Among publicly insured patients, the risk of POU was 2.0% overall and 3.2% vs 1.3% among those with vs without 1 or more initial prescriptions (AME, 2.3 percentage points; 95% CI, 2.1-2.4 percentage points). Among privately insured patients, the risk of POU was 0.9% overall and 2.0% vs 0.6% among those with vs without 1 or more initial prescriptions (AME, 1.3 percentage points; 95% CI, 1.2-1.3 percentage points). The AME of 1 or more initial prescriptions was 1.0 percentage point higher in publicly insured patients (95% CI, 0.9-1.1 percentage points).

**Figure.**
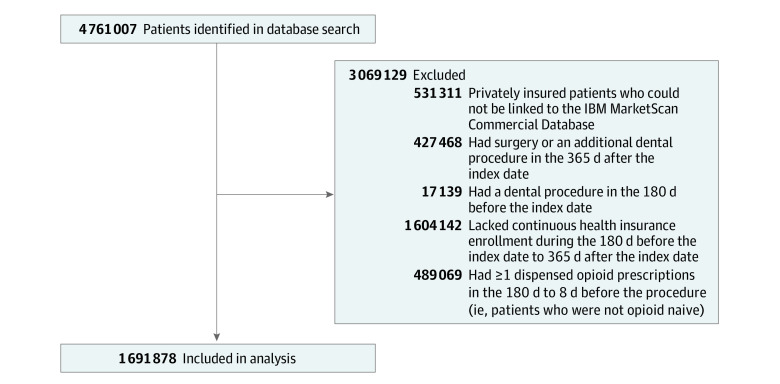
Sample Inclusion and Exclusion Criteria for Study Patients Patients with claims containing Current Dental Terminology codes corresponding to one of 120 dental procedures between July 1, 2014, and December 31, 2017, were included. Procedures were either invasive (eg, tooth extraction, endodontic therapy) or emergent (eg, palliative emergency treatment of dental pain). Noninvasive procedures for which opioid prescribing is extremely rare, such as tooth restorations, were not included. The study excluded patients who had surgical procedures (as defined by anesthesia-related *Current Procedural Terminology* codes) or additional dental procedures during the 365 days after the index date (ie, another claim with a Current Dental Terminology code corresponding to one of the 120 procedures). These groups were excluded to maximize the probability that cases of persistent opioid use were associated with initial prescriptions for the index dental procedure rather than opioid prescriptions for other procedures. Patients with subsequent emergency department visits were not excluded because many such visits are for conditions that would not result in opioid prescribing, such as asthma exacerbations. Patients who had other dental procedures during the period spanning between the 180 days prior to the index date to the 1 day prior to the index date were excluded. This exclusion affected only a small number of patients with index dates in 2014.

**Table.** Risk of Persistent Opioid Use Associated With Initial Prescriptions for Dental Procedures^a^

Exposure or covariate	No. (%)		Adjusted odds ratio (95% CI)^b^	Average marginal effect (95% CI)
	Patients with persistent opioid use (n = 22 059)	Patients without persistent opioid use (n = 1 669 819)		
Initial prescription status^c^				
No initial prescription	11 052 (1.0)	1 150 889 (99.0)	[Reference]	[Reference]
≥1 Initial prescriptions	11 007 (2.1)	518 930 (97.9)	3.16 (3.03 to 3.31)	1.5 (1.5 to 1.6)
Payer type				
Private	9741 (0.9)	1 056 183 (99.1)	[Reference]	Reference
Public	12 318 (2.0)	613 636 (98.0)	2.60 (2.49 to 2.71)	1.2 (1.1 to 1.2)
Age group, y				
13-25	3880 (0.6)	647 164 (99.4)	[Reference]	[Reference]
26-34	4254 (2.0)	213 091 (98.0)	3.11 (2.97 to 3.25)	1.1 (1.0 to 1.1)
35-44	4551 (1.8)	247 107 (98.2)	3.56 (3.41 to 3.73)	1.3 (1.3 to 1.4)
45-54	5049 (1.7)	288 289 (98.3)	4.04 (3.86 to 4.22)	1.6 (1.5 to 1.6)
55-64	4325 (1.6)	274 168 (98.4)	3.87 (3.68 to 4.06)	1.5 (1.4 to 1.5)
Sex				
Male	7671 (1.0)	749 324 (99.0)	[Reference]	[Reference]
Female	14 388 (1.5)	920 495 (98.5)	1.32 (1.28 to 1.35)	0.3 (0.3 to 0.4)
Mental health diagnosis				
No	15 413 (1.1)	1 401 866 (98.9)	[Reference]	[Reference]
Yes	6646 (2.4)	267 953 (97.6)	1.53 (1.48 to 1.58)	0.5 (0.5 to 0.6)
Substance use disorder diagnosis				
No	19 152 (1.2)	1 595 393 (98.8)	[Reference]	[Reference]
Yes	2907 (3.8)	74 426 (96.2)	1.46 (1.40 to 1.53)	0.5 (0.4 to 0.5)
No. of Elixhauser comorbidities^d^				
0	13 761 (1.0)	1 339 526 (99.0)	[Reference]	[Reference]
1	4330 (2.0)	217 215 (98.0)	1.40 (1.35 to 1.45)	0.4 (0.4 to 0.5)
2	2093 (2.8)	73 561 (97.2)	1.66 (1.58 to 1.74)	0.7 (0.6 to 0.8)
3	1012 (3.9)	25 056 (96.1)	2.02 (1.89 to 2.17)	1.1 (1.0 to 1.2)
≥4	863 (5.6)	14 461 (94.4)	2.40 (2.23 to 2.59)	1.5 (1.3 to 1.7)
Procedure type^e^				
Tooth extraction	9961 (1.4)	722 580 (98.6)	[Reference]	[Reference]
Problem-focused limited oral evaluation^f^	8125 (1.4)	560 817 (98.6)	1.75 (1.69 to 1.81)	0.8 (0.7 to 0.8)
Endodontic therapy	2454 (1.1)	214 179 (98.9)	1.20 (1.14 to 1.25)	0.2 (0.1 to 0.3)
Palliative (emergency) treatment of dental pain	452 (0.9)	49 810 (99.1)	1.34 (1.21 to 1.47)	0.3 (0.2 to 0.5)
Pulp capping	147 (0.6)	25 633 (99.4)	1.11 (0.94 to 1.31)	0.1 (−0.1 to 0.3)
All other procedures	920 (0.9)	96 800 (99.1)	0.98 (0.92 to 1.06)	0.0 (−0.1 to 0.1)
Year				
2014	3678 (1.5)	242 534 (98.5)	[Reference]	[Reference]
2015	7138 (1.5)	483 476 (98.5)	0.90 (0.87 to 0.94)	−0.2 (−0.2 to −0.1)
2016	5416 (1.2)	442 017 (98.8)	0.69 (0.66 to 0.72)	−0.5 (−0.6 to −0.4)
2017	5827 (1.2)	501 792 (98.8)	0.66 (0.63 to 0.69)	−0.5 (−0.6 to −0.5)

^a^ Percentages in the second and third columns refer to row percentages.

^b^ Logistic regression was used to model the occurrence of persistent opioid use as a function of having one or 1 initial prescriptions, payer type (0 = private and 1 = public), and their interaction (the interaction term is not shown in the table). Covariates were age, sex, mental health and substance use disorders, number of Elixhauser comorbidities (as a categorical variable), procedure type, and year. Comorbidities were based on diagnosis codes on medical claims occurring during the 180 days before the index date. In assessing the number of Elixhauser comorbidities, those related to mental health and substance use disorders were excluded.

^c^ The MarketScan databases do not contain information on prescriber specialty on pharmacy claims. Following a prior study that used the MarketScan Commercial database to assess the risk of persistent opioid use after wisdom tooth extraction among privately insured adolescents and young adults,^2^ an initial prescription was defined as a dispensed opioid prescription during the 7 days before to 3 days after the index date. The week before the index date was included to account for the possibility that dentists may provide perioperative opioid prescriptions before the procedure even though these prescriptions are intended to treat postoperative pain.

^d^ The mean (SD) number of Elixhauser comorbidities among patients with and without persistent opioid use was 0.7 (1.2) and 0.3 (0.7), respectively.

^e^ The 5 most common procedures in the sample are listed. Regressions included indicators for these 5 procedures and an indicator for a sixth category that included all other procedures.

^f^ This procedure frequently refers to evaluation for acute dental pain.

## Discussion

Dental opioid prescriptions were associated with a 1.0–percentage point higher risk of POU among publicly insured patients compared with privately insured patients. This difference translates to a substantial excess number of POU cases, as US dentists accounted for 11.4 million opioid prescriptions in 2016.^4^ One potential explanation is that publicly insured patients may have higher rates of undiagnosed substance use disorders, a risk factor for POU.^1^ Because of access barriers, including the lack of adult Medicaid dental benefits in some states, patients may be prescribed opioids instead of receiving definitive dental care.

Our study has some limitations, including the lack of patient location information in Medicaid claims. Dental opioid prescribing rates vary regionally.^5^ Estimates would be confounded if the risk of POU similarly varies regionally, although we are unaware of data supporting this possibility.

Our findings suggest that studies of the privately insured underestimate the risk of POU associated with dental opioid prescribing.^2,3^ The results further highlight the importance of avoiding dental opioid prescribing when nonopioids provide effective analgesia, which is the case for most dental procedures.^6^ More broadly, prior studies of POU have largely excluded publicly insured patients.^1,2,3^ If the findings of this study are generalizable to other procedures, the risks of perioperative dental opioid prescribing may be greater than previously appreciated.
